# A systems thinking approach for examining the turning points in the Finnish public sport policy

**DOI:** 10.3389/fspor.2025.1591211

**Published:** 2025-05-22

**Authors:** Kati Lehtonen, Petri Uusikylä, Harri Jalonen, Sari Lappalainen

**Affiliations:** ^1^ School of Health and Social Studies, JAMK University of Applied Sciences, Jyväskylä, Finland; ^2^ School of Management, Social and Health Management, University of Vaasa, Vaasa, Finland

**Keywords:** sport policy, systems thinking, complexity, timeline mapping, turning point

## Abstract

**Background and study aim:**

This study proposes a longitudinal analysis of the Finnish public sports policy and explores the dynamics between turning points from a systems thinking perspective. We argue that a better understanding of the dynamics and relationships between turning points, interruptions, and new paths in the policy process can guide future options for public sports policies.

**Material and methods:**

Documentary and interview-based materials were used as data and timeline mapping as an analyzing method.

**Results:**

Our results show that there have been four turning points during the review period. The first turning point in the early 1990s is fundamental and it had large-scale effects on the direction of sport policy. The other three are moderate in their nature, smoothly affecting the direction of the policy process. Policy venues have changed from theme-specific working groups to permanent entities operating within the state administration. At the same time, party politics has returned to politics, a phenomenon that seems at odds with the first turning point.

**Conclusions:**

For decision-makers and practitioners, the current policy space requires increasing ability to make decisions on a long-term basis despite several interests, increasing complexity of governance and the needs of differentiated subsystems.

## 1 Introduction

There is an enduring societal fascination with sports, and it intensifies the expectations of public policy regarding the strategies, goals, and methodologies employed in the implementation of sports policies. Physical activity (PA) is recognized as a national asset, and the state of the nation's health is closely intertwined with economic, medical, cultural, educational, and social accomplishments (1). This connection between sport policy and public policy compels stakeholders in sports policy to provide a more comprehensive demonstration of the efficacy and dynamics of their policy initiatives. In addition, the direction of development, where the nature of government interventions for sports is broadening as well as the levels of public funding, is still relevant [cf. (2)].

According to the World Health Organization, WHO (3), policy actions have been insufficient and government strategies for increasing PA have not consistently increased the proportion of the population meeting recommended levels of activity. In response to this lack of progress, there has been a growing recognition of the role of systems thinking in helping to frame responses to complex public health challenges (4, 5). The systems thinking approach to policy change highlights the underlying conflicts, path dependencies, and turning points that enable the system's transformation (6, 7). According to Midgley and Rajagoplan (8) systems thinking refers to the practical application of systems ideas to address or prevent complex environmental, social and organizational issues.

In this research we apply systems thinking approach to examine public sports policy which, according to Howlettś and Cashoreś (9) definition of public policy, is whatever a government chooses to do or not to do. Houlihan (2) has related public policy to resources, and stated that “public policies are those actions that originate within, or are dependent upon resources of, the state”. Based on this, we define public sports policy as a set of vision and objectives, necessary legislation, strategic directions, roles, resources, and responsibilities, and sport policy actions for the development of a specific country. Our empirical analysis centers on Finnish public sport policy and we focus on the turning points, namely moments or events that lead to a change in the direction or behavior of a system [cf. (10)]. This can be due to internal changes within the system or external influences that push the system beyond its current state. To better understand the dynamics and the interconnected nature of the turning points, interruptions and new paths of the sport policy process can be constructed and predicted.

Our contribution is twofold. First, we enhance existing sport policy research although systems thinking and related methods have not been used extensively in research on public sports policies. The systems thinking approach applied to public health or national physical activity research is already familiar to some extent [e.g., (5, 11–13)], but it is in the early stage of development, with a preponderance of descriptive approaches and a dearth of complex analysis (14). The systems approach is also generally familiar when researching for example sports injuries, coaching, and sports science (15, 16). In addition, the complexity framework has been used to examine sports organizations as complex systems (17) and the development of professional sports leagues from a socio-political perspective (18).

Second, we offer policymakers perspectives that better enable them to understand the complex nature of sports policy. We lean on the real-world problems of public policy, where stakeholders and policymakers need to better understand the nature of complex phenomena, such as climate change or obesity (15, 19). This perspective is also familiar in the European Union (20), where sport is recognized as a fast-moving policy area. The importance of sport is not only obvious for health and wellbeing reasons but also for several key areas, such as social cohesion, territorial regeneration, and economic growth.

What makes the Finnish public sports policy a fascinating object of study is that its characteristics can be associated with unstable and uncontrollable attributes. Public sports policy has included breakthroughs during the last three decades. As a result, the main policy agenda has varied from one side to the other, for example, between elite sports and sports for all. This observation indicates that policy subsystems and actors have been able to break down policy monopolies, and many interests have intervened in public sports policy [cf. (21, 22)]. Since it is known that PA and elite sports are generally the pillars of national sports policy in different countries, the study also offers a comparative perspective internationally.

In addition, the reform of institutional structures of Finnish sport policy has appeared to be a continuous spiral. It has been determined by adapting sports policies to changes in the paradigms of public administration, such as New Public Management (NPM) (23). In the early 1990s, when NPM fuelled the Finnish administration, various changes were reflected in the state's stricter resource-based management. This transition also changed power relations and formed new policy venues and institutional structures [cf. (24)]. As a historic ground flow, Finland's public sports policy has also been influenced more by party politics than in other Nordic countries; the entire sports system was built on it until the early 1990s [e.g., (25, 26)].

Based on these perspectives, the aims of the study are (1) to empirically identify the turning points in Finnish public sport policy starting from the 1990s to today, (2) to analyze the dynamics and interconnectedness between the turning points, and (3) to consider how the systems approach helps interpret and understand longitudinal policy changes. The main data of the study consist of sports policy working-group memorandums (*n* = 23) and group interviews with government officials (*n* = 5). In addition, we use existing studies to frame and contextualize the analysis. The research method used is timeline mapping.

The remainder of this paper is organized as follows. We first describe the basic elements of Finnish sport policy to increase the understanding of our empirical context. After that we consider the core points of systems thinking in relation to the theories commonly used in policy analysis. Next to that we describe the research design. The detailed findings of our study are provided in the Results section, after which discussion together concluding remarks are presented.

## 2 Basics of Finnish public sport policy

Activities based on civil society and non-governmental organizations such as National Sport Governing Bodies (NSGBs) have historically been characteristic of Finnish sports policy. This was because civic movements generally promoted many social issues before the country's independence in 1917. Another significant feature in the early stages of sports policy was that the sports organizations of civil society were politically divided because of the 1918 Civil War even so that local sport clubs were members of either the left- or right-wing central sport organization. The birth of a deliberate public sports policy occurred just after the 1918 Civil War, and the first administrative board for sports, currently called the National Sports Council (VLN), was established in 1920 (27).

The Ministry of Education and Culture (OKM), currently the state administrative organization responsible for sports, was chosen for its leading role in the late 1960s (ibid.). From the beginning, the main tasks of the public administration of sports have been resource allocation and the steering of national sports policy based on laws and parliamentary decisions. According to OKM (28) “sport policy is designed to promote sport and physical activity and, through them, the wellbeing of the population, as well as competitive and performance sports and related civic activity”. The state's sports budget is approximately 150 million euro, from which subsidies are targeted mainly to national sports organizations such NGBs (40–45M€), sports facilities (30 M€), and sports institutes and municipalities (20 M€ each) (29).

The first administrative board, the National Sports Council, serves currently as a panel of experts assisting the Ministry of Culture and Education appointed by the government for the parliamentary term. The Council is called upon to address major issues of fundamental importance related to sports and to evaluate the impact of government action in the field of sports. Regarding legislation related to public sport policy, the Act on the Promotion of Sports and Physical Activity (390/2015) (30) came into force for the first time in 1980. This law promotes PA and elite sports, as well as the responsibility and cooperation between the state administration and municipality, state administrative bodies, and state funding in the field of physical activity. According to the Act (4§), “the Ministry of Education and Culture is responsible for the overall management, coordination and development of the national sports policy” and “when performing the duties defined herein, the State shall, as appropriate, engage in cooperation with municipalities, non-governmental organizations and other actors in the field of physical activity and sports”. In its current format, the law creates the basis for a pluralistic system, not as attached to the activities of NGOs as in other Nordic countries.

## 3 Theoretical background

Systems thinking has largely developed as a field of inquiry and practice in the 20th century and has multiple origins in disciplines as varied as biology, anthropology, physics, psychology, mathematics, management and computer science (31). Senge (32) has stated that systems thinking is discipline for seeing wholes and a framework for seeing interrelationships rather than linear cause-effect chains. He continues that processes and patterns of change are those under consideration rather than static snapshots or individual components in isolation.

Overall, systems thinking provides a holistic perspective that helps in identifying the dynamic nature of systems. Following Meadows (6), this kind of thinking allows us to identify root causes of problems and see new opportunities, for example, in the progress of public sport policy. In policy research, the systems thinking approach focuses on observing changes in system dynamics and recognizing patterns within policy processes (7, 33). From the viewpoint of our study, the following elements of the systems thinking are crucial: the nonlinearity of the policy process, relations between the turning points and the complexity evolving during the process (34).

The basic elements of systems thinking are opposite to the reductionist mindset which has been the mainstream when governing, practising or viewing the sport (16). The complex nature of sport also requires new approaches to analyze sport policy, where for example power relations, resource dependencies, historical roots, beliefs, and structural aspects are under consideration at the same time (35). Currently, the complex nature of sports is having growing interest overall (16) and also in sport policy issues (36). Sam (37) points out three characteristics of complexity that can be applied to sports policy: (1) difficulties in issue definition, (2) uncertainties regarding causal chains and working mechanisms, and (3) a propensity for remedies to result in new or unintended problems or exacerbate existing challenges. When these characteristics are applied to policy actions and their implications, a systems thinking approach suggests that the nature of a problem cannot be understood separately from its solution (36).

While the use of the systems approach within one segment of sports policy research (i.e., PA) is growing [see (14)], it is minimal for examining public sports policy as a whole or understanding historical roots of policy processes. The results of a systematic review on the development of sports policy research during the years 2000–2020 show that the systems approach was not used as an analytical framework for researching sports policy, even though there has been growing interest in the new theorizations of sports policy among researchers in the field (1). Jayawardhana and Piggin (38) have suggested that developing a unique framework for analyzing sports policies could be one option. Overall, they call for a holistic approach to sports policy analysis after examining the pros and cons of the widely used frameworks in sports policy research, such as the Advocacy Coalition Framework (ACF).

The key differences between change theories lies in the nature and the degree of the dynamics of change (39). Change can be gradual and incremental; the chosen policy is an intentional muddling process (40). Overall, in the studies of policy change, particular attention has been devoted to micro conversion (41), policy coalitions, and shared belief systems (42), as well as hidden agendas and invisible power structures (33). Policy feedback theory (PFT) suggests that public policies, once implemented, influence future political processes and behaviors (43). Policies shape individuals' and groups' perceptions of government and its political efficacy and thus influence political participation and interest group formation. “Feedback effects” mean that policies are not just outcomes of political actions but are also catalysts for future political changes (44). By reshaping the political landscape, policies can create self-reinforcing cycles, making certain future policy outcomes more likely.

The aim of the Advocacy Coalition Framework (ACF) is to establish a systematic connection between the macro perspective (which comprises factors like the socio-economic environment and public opinion) and the “meso” viewpoint (which focuses on policy creation within a single policy subsystem). The ACF outlines four distinct routes for achieving policy transformation, namely, external influences, internal occurrences, policy-focused learning, and negotiated agreements (24). These pathways highlight the complex interplay among the factors that drive changes in public policy.

According to the basic idea of the path dependence theory (PDT), later events in the event chain are dependent on earlier events (45). Breakthrough stages, meaningful moments and critical turning points are important from the point of view of the progress of the process. The structural, social and economic choices made during them guide the direction of the process. These turning points are often accompanied by interventions that influence decision-making during the process (44).

Path dependence is related to the punctuated equilibrium theory (PET), which is developed by Baumgartner and Jones (46). PET seeks to explain the dynamics of how and why certain issues rise to prominence on the political agenda, while others decline or remain stagnant. Social or political change takes place in time as an alternation between quite short but strong periods of change and steady change. When looking at path dependence, both change and permanence are thus perceived (21).

When comparing the main points of the above-mentioned theories with the core idea of systems thinking, PDT comes closest, especially as both deal with understanding how historical events and interactions shape the behavior and evolution of complex systems [cf. (47)]. Path dependence highlights how past choices constrain future options, while systems thinking considers how these historical influences interact within the system [cf. (48)]. Both PDT and systems thinking identify turning points as moments of change; path dependency theory focuses on critical moments in the development of an individual or system, while systems thinking focuses on strategic points where changes can have a broader impact on the system.

This distinction is also the basis for our choice to examine the turning points in Finnish sports policy in the framework of systems thinking. In our view, applying systems thinking approaches to the longitudinal analysis of public sports policy requires understanding the interconnectedness between turning points, which makes it possible to interpret the development and dynamics of sports policy more holistically. Using the concept of a turning point aims therefore not to identify the radical moments of change such as commonly used concept focusing event on policy studies (22), but rather broader changes in the direction of the policy and how the patterns under consideration have influenced this new direction. This is the case also in the framework of a systems thinking, where turning points show the change in a broader direction while for example breaking points or critical points indicate more sudden changes [see (6)].

## 4 Materials and methods

This study used the state's sports policy working-group memorandums (*n* = 23) and interviews with state officials (*n* = 5) as research data. Our research covers the years 1990–2023. The beginning of the research period is based on previous research, showing the changes in institutional structures and political-administrative practices of Finnish public sports policy. The role of NGOs in public sports policy decreased, and the state administration, especially civil servants, became significant power users (23, 26).

We collected all the working groups memos which occurred during the research period and focused on elite sports (*n* = 6), PA (*n* = 3), funding (*n* = 6), law (*n* = 2), and general sports policy issues (*n* = 6). All working groups have been appointed by the ministry responsible for sports at the time. The memos are listed as part of the References section, and in the Results section, they are referred to by the codes M1–M23.

The analysis of the working-group memos and current research was conducted by applying the timeline mapping method. It is also methodologically relevant when the aim is to understand an issue's landscape and history and how contextual factors such as sport policy actions, practices, resources, and laws were located in a certain temporal dimension (49). In addition, it helps to organize the data so that considerations, how the focus of actors or larger systems are shifting over time or sports policy principles evolves, are possible (50).

The working platform to organize the data was Excel software, where the material was structured line-by-line in relation to the policy agenda, policy venues, the balance of parliamentary politics (macro politics), institutional structures and arrangements, and the governance paradigm, which refers to the general operational logic of public administration prevailing in the research period, such as NPM. Of these elements, the agenda describes the main lines of public sports policy, i.e., the division of sport for all and elite sport, and their mutual change. These agendas can also be considered as subsystems of public sports policy. The chosen framework and five elements were developed as a theory driven and based on earlier research. Policy agenda is a set of issues which are prioritized during the policy process and thus at the core of policy analysis (51). In this research the agenda was connected to policy venues, because agendas of public sports policy have been produced over the years in several working groups and other bodies appointed by the administration, where consultation of stakeholders and a common political will are realized. These entities are examined as policy venues. Macro politics provides the basic lines of public policy and is more of an external influence on public sports policy. However, in the context of this research, the element of macro politics is crucial, because of the historical roots of Finnish sport where right- and left-wing politics have had strong impact (23, 52). Institutional practices and structures as well as governance paradigms, in turn, provide the framework for the logic by which public policy is carried out.

When analyzing the data, the state's sports policy working groups as policy venues were divided annually on a timeline to show frequent event series. Similarly, in the analysis of the first phase, the main policy agendas and practices, such as changes in legislation, were compiled from the working-group memos mentioned above. The parliamentary balance of power was marked on the timeline every year so that the Prime Minister's Party and the Sports Minister's Party were included. In the first phase, an initial analysis of the policy process was conducted based on documents and earlier research. When we analyzed the extent or intensity of change, we defined a more significant turning point as a result of several simultaneous changes. An example of this is the shift in institutional structures and governance paradigms that influenced the direction of the policy process in many ways in the early 1990s.

Group interviews with state officials were conducted in the second data collection phase in January 2024. The interview was also a first-stage validation, and a process part of the timeline analysis. Officials were selected based on their positions in the Ministry of Education and Culture. The interview call was sent to all officials (*n* = 8) of whom five participated. The group interview lasted 75 min, and it was recorded. Before the interview, the initial analysis was presented; it formed the basis for discussion. The idea was to either confirm or refuse the initial results and create new interpretations which could not be found from the documents as Lowry and Mullins (53) have stated.

One of these themes was the power dynamics of subsystems and actors maintaining them, which are not mediated by documents but are essential when analyzing policy processes (21). As examples of concrete questions, the following were asked: Do you agree with the temporal dimension of the turning points (identifying turning points)? Where do you think the contents of sports policy are negotiated (policy venues and agendas)? How has cooperation between officials and politicians changed (political-administrative practices and power dynamics)? Have general changes in policymaking or some practices impacted the ways of making public sports policies (macro politics)?

In the third phase, both materials were combined, based on which a final interpretation of turning points in the policy process was made. A summary of the data collection process and analysis is presented in Table 1.

**Table 1:** Phases to collect and analyze the data.

Project phases	Project activity
Phase 1	• Collecting sport policy working-group memos (*n* = 23) and adding them yearly to the Excel file based on working years. • Memos consist of the following themes: overall sport policy (6), funding (6), elite sport (6), PA (3), and law (2). • Compiling desk review on current literature and working-group memos.
Phase 2	• Making an initial timeline analysis in an Excel file.
Phase 3	• Workshop and interviews with state officials (*n* = 5) and presenting the initial results and observations to discuss. • Compiling timeline based on the workshop.
Phase 4	• Analyzing data sets to form a final timeline and results.

## 5 Results

Four distinct turning points were identified during the research period (Table 2). Below, we provide a more comprehensive explanation of the main findings regarding these turning points. Subsequently, we analyze the theoretical contribution of the research and carefully evaluate the added value of applying a systems perspective.

**Table 2:** Turning points and changes in selected elements.

The temporal occurrence of turning points	Paradigm of governance	Institutional structures and arrangements	Parliamentary equilibrium of power	Policy venues	Policy agenda
1990–1993	Transitioning to NPM, corporatist negotiation practices are decreased. Politics out of policy.	The central government's role grows as the director of sports policy, and NPM thinking produces a new, decentralised, multi-powered structure of sports.	Stable	From committees work to working groups.	Funding for sports organisations and criteria for performance guidance.
2006–2007	NPM prevails, as does performance management implementation.	Stable	Stable	Different venues are activating.	Centralisation of elite sports structures and decreasing the number of national sports organisations. Children’s physical activity.
2013–2014	Transition NPM to New Public Governance (NPG), increased number of administrative coordination and management levels and political guidance.	An independent unit is established for elite sports.	Instability: cycles of the balance of power in party politics are shortened.	Latent.	See above.
2020–2023	NPG dominates; the political guidance and the representation and impact of interest groups continue to increase. Politics into policy.	One central sports organisation for NGOs. Public funding for sport is no longer earmarked; budget processing is politicised. The government programme includes an independent programme for promoting physical activity, as well as additional funding.	Unstable.	Temporary working groups are replaced by established ones: Cross-administrative coordination body. Ministerial working group to supervise the implementation of the Government Programme. The National Sports Council continues as a formal political body.	Physical activity or even, conversely, inactivity.

### 5.1 Turning point 1990–1993: fuelled by NPM and new institutional structures

The first turning point at the beginning of the 1990s was the end of a long period of corporatist negotiation practices and the reign of party-politically divided Finnish sports movements, starting in the first decade of the 1900th century [e.g., (23, 52)]. Structurally, the sports system was dispersed, and two politically committed NGOs were replaced by four national organizations responsible for the promotion of different fields of sports policy (elite sports, youth sports, sports for all and one new central sport organization).

The catalyst for the change was the right-wing central sport organization's financial difficulties and NPM thinking, which crosscutted public administration. The NPM, adopted extensively in Western countries, emphasizes productivity, efficiency, and accountability (54, 55). At the national level, the key administrative reform projects include resource-based management, state-subsidized reform, and the corporatization of state enterprises.

In the case of NGOs, resource-based management was implemented to direct the activities of these organizations, and the state defined key result areas and criteria for measuring and evaluating results (M1,2). The idea of NGOs acting as service producers also brought market-based operating logic to the sports movement [see (54)]. Simultaneously, policy venues underwent a change, and the work of extensive committees was transferred to restrictive thematic working groups. This change decentralized the venues in which the sports policy discussions took place. The National Olympic Committee oversaw the development of elite sport (M7−8), and the Ministry of Social Affairs and Health nominated working groups to promote PA policies (M13).

In terms of policy content, the sports policy agenda was emphasized by the preparation of subsidy criteria for sports organizations, which remained the main agenda of the working groups for several years after the first turning point (M1–6). Before the turning point, the corporative practices defined the relations between the state and sports stakeholders, namely party-political based NGOs, but the new institutional structures and arrangements emphasized the growth of the administration's power, which can be described as a tendency to take “politics out of policy” [cf. (56)].

### 5.2 Turning point 2006–2007: activating venues and setting the agenda

The second turning point in the research period was mainly determined by the activation of policy venues in a wide range of policy agendas, including elite sports (M11, 12), PA (M14), and funding (M5). In addition, the overall strategy of sports policy was discussed in principle for the first time since the 1990s (M18–20). According to the interviews, redefining the content stemming from the turn of the 1990s was necessary:

Subsequently, when the continental plates changed in the 1990s, we were in a constant state of change. We have been looking for a new direction but have not been able to define this common will. In addition, NGOs have been struggling to seek direction and autonomy.

In addition, according to the interviews, the proposals of the working groups, such renewing elite sport coordination, were also “weakly implemented”. At this turning point, elite sports were set as the main agenda because there was a need to carry forward the findings of the 2004 Report (M4). The government allocated resources to implement elite sports reform between 2009 and 2012 to increase the success of national elite sports. The practices pointed out were the centralization of elite sports system structures and their management practices (M11,12).

When comparing this turning point to the previous one, the difference is that the NPM paradigm can be considered an established administrative paradigm, and several operating practices have been created to implement it within sports policy. The institutional structures and methods of steering sports policies have also been balanced. These changes were not fundamental and did not immediately lead to structural reforms as in the previous period. Thus, the turning point focused on activating policy venues and creating a new agenda for sports policies.

### 5.3 Turning point 2013–2014: new administrative paradigm and weak signals of politicization

At this turning point, the most essential factors culminated in the objectives set as the policy content of elite sports becoming concrete as a new institutional structure. An independent high-performance sports unit was established in 2013, and the state became more distant from the leading elite sports policies; the task was delegated to the National Olympic Committee. In this respect, the joint management system of the state and NGOs was clarified (23). However, the reform did not end in 2013; five years later, the reformed Olympic Committee took responsibility for elite sports and sport for all activities, which meant a significant expansion of operations from previous activities focused on elite sports. From this perspective, the turning point was a catalyst for centralizing the institutional structures of civil society stakeholders in sports.

The general factors behind public sports policy were significantly affected by the gradual transition from NPM to New Public Governance (NPG) thinking, where networking became a new way to operate both within the state administration and between the administration and various stakeholders [cf. (57)]. This was particularly evident in the promotion of PA policies. The number of network-based national large-scale programs for PA increased, and the state and public institutions such as schools began to appear as a crucial player in this policy subsystem (58). A change in legislation supported this direction (M14); the revised Act on the Promotion of Sports and Physical Activity (390/2015) (30) no longer defined NGOs as the primary actors for organizing sporting activities for the population.

According to the interviews, the turning point was also accompanied by silent signals about the increased use of power by the minister's cabinet, and the number of the special assistants of the minister began to increase, as did the number of direct contacts of stakeholders with the Sports Minister's cabinet, as a state official described:

It was at that time when they [stakeholders and interest groups] started to call the Sports Minister or assistants directly. They undergo a normal process in which decisions are to be prepared. Now, we follow what is happening because of this fast-lane policy.

A macro-level phenomenon related to the power dynamics of parliamentary party politics also occurred at this turning point. During the first two decades of the period under review, the Prime Minister's Party and the Sports Minister's Party were in power for four years, and the overall appearance of politics was balanced. Since this turning point, instability was the most characteristic feature. In particular, the ministers responsible for sports changed more often than before; in one year, there could be up to two different ministers from two different parties.

### 5.4 Turning point 2020–2023: politics into policy

The final turning point can be described as a return to the early 1990s by looking at the extension of the changes that took place simultaneously in institutional structures, venues, and agenda-setting. The administration and its governance practices were organized in a multilevel manner so that state officials began negotiating sports policy on the cross-administrative coordination body established in 2020 (59). Similarly, the ministerial-led working group began to monitor the implementation of sports policy practices recorded in the valid government program.

In practice, these were issues related to PA promotion with extra funding of approximately 20 million euro per year [see (60)]. In addition, the National Sport Council, which had been an established structure for a long time to evaluate sport policy [see (61)], began to appear as a venue of advocacy for individual interests as one of the state officials described: “the wider issues of sport policy are not in the Council's agenda, I feel they are lobbying even single projects”.

In the last turning point, the venues changed so that temporary working groups consisting of stakeholders and administration representatives were replaced by long-term venues mainly consisting of government officials and politicians. Simultaneously, public administration operators and politicians took a stronger approach to policy practices promoting PA. It can be said that PA policy actions have been governmentalized, implying tighter state guidance, political interest, and the growing role of public institutions as implementers of policy actions.

Correspondingly, elite sports, which had a strong agenda at the beginning of the 2010s, could no longer fit into the key agendas of sports policies or policy venues. In the interviews, state officials did not recognize the venues where elite sport is negotiated; instead, they considered, “Does anyone do elite sport politics?” Thus, it can be stated that the hierarchical balance between the main agendas and subsystems, that is, elite sports and physical activity, clearly changed compared to the turning point in 2013–2014.

### 5.5 A synthesis of the dynamics between the turning points in the framework of systems thinking

**Figure 1:**
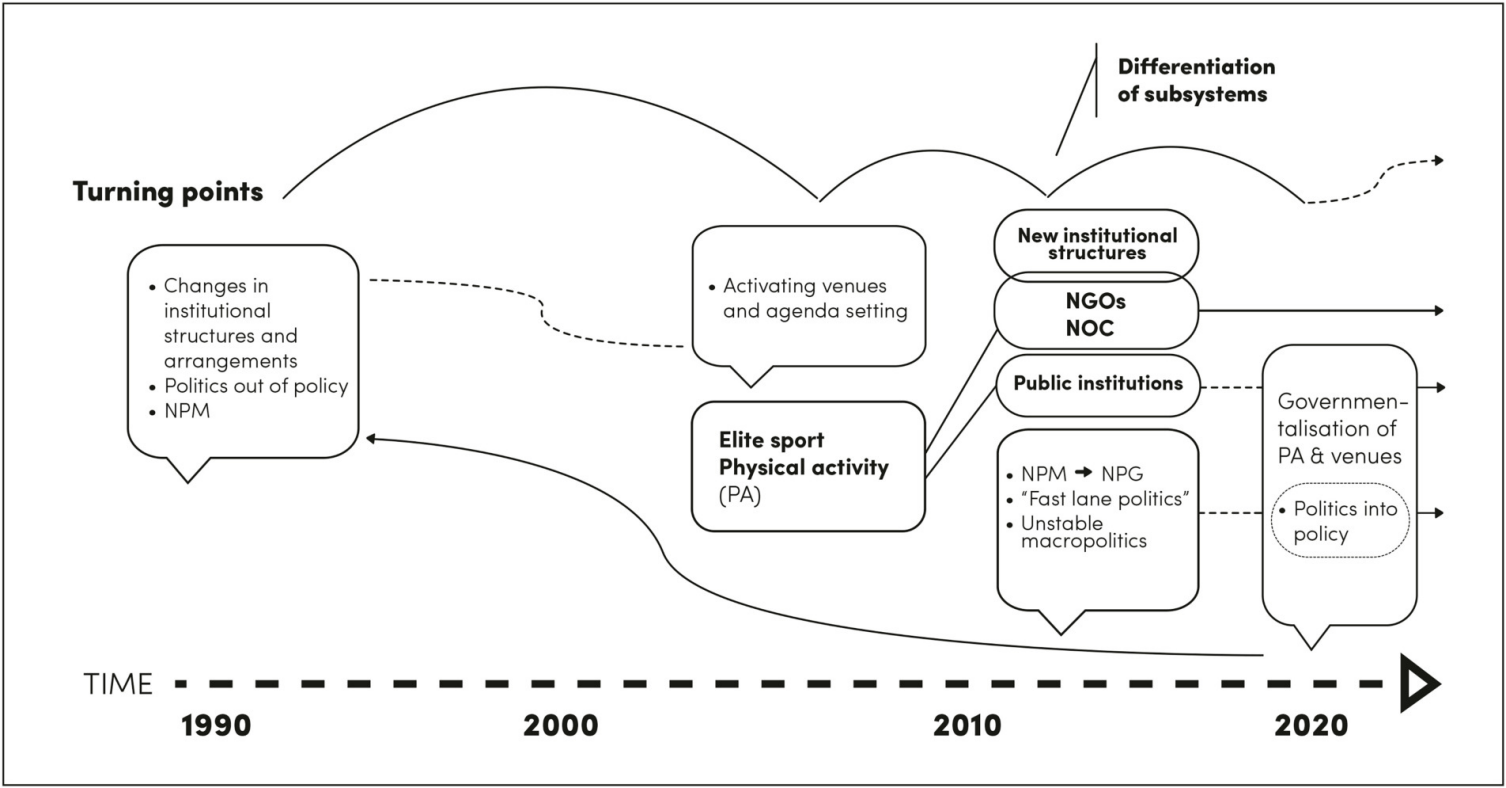
The summary of the temporal dimension between turning points and the key elements of each turning point.

Examining the dynamics between the four turning points from systems thinking perspective reveals that the turning points differ in nature (Figure 1). The timing and tempo between the turning points were also asymmetrical. The difference between the first and second turning points is almost 20 years, after which turning points occur more frequently.

This study defined the first turning point based on previous studies, where Finnish sports policy underwent a fundamental change in institutional practices and the paradigm of public administration. However, based on the results of this study, the turning point in the 1990s was much more, as it caused a permanent non-equilibrium in public sports policy.

The two following turning points were more moderate in nature. They subtly influenced the direction of the process but were not radical turning points [cf. (6)]. The general impression is that the direction of the process in public sport policy has been rather sporadic and not strategically driven after the first turning point. Although the last turning point covered several simultaneous changes, similar to the first turning point, it was about a continuum based on path dependence rather than a consciously implemented or chosen. This is in line with Cilliers (47) who argues that complex systems have a history or path dependency, where decisions and actions made previously influence here and now.

The last turning point resulted from the gradual growth of politicization and the setting of one subsystem of sports policy as the central goal of public policy. Permanent administrative negotiation arenas were built around PA agenda, but the faster cyclical nature of macro-policy was more of a general policy change than a specific feature of sports policy. The growth of politicization can also be seen as a change in power dynamics. During the reviewed period, a clear cyclic transition was observed in how party politics returned to influencing administrative practices, that is, politics returned to policy.

If turning points are viewed from the perspective of bifurcation, where option for a one new path is offered (62), we may conclude that a new direction has been taken in Finnish sports policy. Attention must be drawn to the turning point in 2013–2014 when the differentiation of subsystems (elite sports and PA) occurred. This solution has led to a situation in which elite sports have been encapsulated as an independent sports policy subsystem managed by a non-governmental actor, the National Olympic Committee. PA has been governmentalized as the main agenda of public sports policy.

In the future, this path may lead to the detachment of elite sports from the policy agenda. This alternative direction is contradictory because public money is still invested in elite sports, and governments have to take a stand on global crises and wars, due to which elite sports, traditionally considered autonomous, becomes tighter part of international politics between countries [see (63)]. Another option and future path could be for the state to participate more than currently in defining the contents of elite sports, as it does in the PA subsystem.

Public administration paradigms affect both the institutional structures and ways of implementing policies. In the first phase, the radical transition to NPM doctrine-based governance changed the institutional structures and practices of NGOs. Later, the growth of networking and the application of NPG were slow and most visible in national programs, which also involved the state as a partner and the role of a financier. According to Lapsley and Miller (64), these gradual transitions of administration and even the overlapping of paradigms are typical, which means that the administration mechanisms do not exist only in certain periods. In general, increased networking, politicization, and multi-level governance have increased the complex nature of sports policy at the end of the review period.

## 6 Discussion

This study examined Finland's public sports policy from a systems thinking perspective. The finding underscores the varied nature of change in determining a new policy direction and the significance of turning points as occasions when a new path is formed (62). Overall, the analysis of turning points revealed the temporal nonlinearity of policy formation and variation in the power of change from gradual transitions to radical transformations (39).

This study contributes to public policy research by first applying systems thinking to observe changes in system dynamics and recognizing patterns within policy processes. A notable finding is the transition from “politics out of and into policy”. In the early 1990s, efforts were made to reduce political power by enhancing the role of civil servants; however, at the latest turning point, the roles of ministers were strengthened, reinforcing party politics and political decision-making both in policy arenas and at the actor level. This politicization was not an intrinsic product of sports policy but rather an adaptation to broader political trends. The findings of the study illustrate the simultaneous fragmentation of subsystems and the segmentation of one policy area, i.e., PA, into others. Nested and interconnected policy clusters can result in “liquid” or hollow policy domains with ambiguous boundaries, obscuring accountability structures and allowing policies to shift beyond democratic oversight.

Second, this study enhanced public policy research by providing a deep systematic analysis of public sports policy. Elite sports and PA have traditionally been the central pillars of sports policy and have been identified as the main subsystems (65). A growing global concern about low PA levels has led to national programs improving this situation (5, 13). The WHO's (3) commitments highlight the complex nature of promoting PA and may guide governments to take broader responsibility in this area. This study revealed similar trends in Finland, particularly regarding the government's approach to integrating guidelines into sports policy procedures. We refer to this phenomenon as governmentalization, which indicates a strong commitment by public institutions to promote physical exercise through government funding and programs. This progress in one subsystem led to structural changes in policy arenas, transitioning from temporary theme-specific working groups to permanent entities within the state administration to engage other ministries and administrations in PA policy practices.

Third, this study offers policymakers insight to better understand the complex nature of sports policy and its practices. Various interest groups and stakeholders' formal opportunities to influence and implement sports policy have diminished, as policy venues primarily comprise ministers, officials, and politicians. If these practices continues, the future path of Finnish sport policy may deviate from the principles of Nordic governance, in which stakeholders and public opinion have historically influenced political forms (66). This direction likely increases the use of hidden power and lobbying and may weaken stakeholders' commitment to policy decisions (67). The situation may also lead to partial optimization of single interest groups' intentions, which supports the traditional reductionist approach in sport governance [cf. (16)].

The result of increased complexity is that the expected outcomes of sport policy are also more difficult to predict. Although solving wicked problems is impossible (68), public sector organizations can develop strategies for managing them effectively. According to Ashby's law (69), a system must possess a range of sophisticated responses to effectively handle the challenges its environment poses. In the context of our research, this means that the state has an increasingly important role in leading public sports policy and bringing different stakeholders together. This approach is even more important if the subsystems of sports policy become differentiated from each other, as we observed.

Every study serves as a gateway to further enquiries; this study is no exception. Public sports policies are likely to increasingly align with international policy strategies, further complicating and in increasing the complexity of the policy landscape. Whether the subsystems of sport policy are becoming differentiated globally, and how global institutions such as the WHO influence the shaping of the complexity of public sports policy nationally, deserves further research. The independent development of single policy subsystems, such as elite sports or PA, from a systemic perspective would also be valuable.

Finally, before concluding, we will consider some limitations arising from the study's data and methods, as well as research ethics issues. The analysis of the dynamics between turning points from a systemic perspective was based on timeline mapping method and materials used included sports policy working-group memos and interviews. In addition, previous research guided to define the research period. In this case, the method used is essential from a synthetic standpoint. The idea behind the timeline mapping method is the chronological arrangement of materials by topic and their co-interpretation with the actors involved in the issue. The latter point was discovered through interviews, which enriched and validated the examination of the phenomenon (49).

Working-group memos provided insights into the incidence and content of policy arenas, whereas interview materials complemented the overall picture and minimized the researchers' too general interpretation of data. A specific example is the change in the functional role of the National Sports Council at the last turning point, which is not detectable through memos alone. This result underscores the benefits of combining different types of materials and highlights the methodological core of systems thinking; the dynamics of a system can only be fully understood by observing phenomena from multiple perspectives (6). Timeline analysis has also been found to strengthen the role of informants and give them the opportunity to share their own interpretation of the process, regardless of the content of other data. This in turn reduces researchers' potential misjudgments and biases related to the topic (70).

We recognize that collecting interview material from sport stakeholders such as national governing bodies of sport would have offered diverse viewpoints, while a more focused interview approach allowed for a deeper exploration of specific topics as Lowry and Mullins (53) have guided. Our starting point in limiting the interviews was that we emphasized the view of the administration, which has played a significant role in Finland. It is evident that this choice may result in sampling bias (71) and, for example, in a group interview, informants may have left something uncommented. On the other hand, in a timeline analysis, shared experience and discussion of the topic is essential and needed (49).

Based on previous considerations, the following points should be highlighted regarding research ethics: the identification of interviewees, the open access of the materials, repeating the research design, and reliability. The latter has been discussed above, especially from the perspective of the number of informants. Since there were few interviewees, it was not appropriate to describe more detailed background information to guarantee their anonymity. Working group policy memos are publicly available, which makes the repetition of the research possible from the perspective of the materials. In addition, describing the research design as accurately and step by step as possible not only enables us to consider reliability of the analysis but also allows the method to be applied in other research aimed at retrospectively understanding the connections between the turning points of certain policy domains [cf. (72)].

## 7 Conclusion

As a concluding remark we underline that systems thinking is not a panacea, but useful when identifying key blockages and considering the long-term consequences of policy actions as Glenn et al. (73) have stated. We underline the idea of Rigby et al. (13) who argued that examinations on how to support system-wide change in the development and implementation of complexity in sport are needed. Even though systems thinking has already been applied in sports research, many perspectives remain to be explored in sports policy. Therefore, as a summary, we rely on Hoekman and Schreeder (36), who have stated that using systems concepts and approaches provides opportunities to rationalize aspects of existing practices and offer insights for improving public sport policy and its research.

## Data Availability

The datasets presented in this article are not openly available because the interview materials have been compiled for the research project and for the use of the project's researchers. The policy documents are not archived, but can be found as online publications. Requests to access the datasets should be directed to kati.lehtonen@jamk.fi.
